# Potentially toxic metals in small ruminant tissues: multivariate analysis and health risk assessment via Monte Carlo simulation

**DOI:** 10.1038/s41598-025-33838-2

**Published:** 2025-12-27

**Authors:** Alireza Fathi-Beyranvand, Azadeh Rashidimehr, Elaheh Askari, Fatemeh Esfarjani, Fatemeh Mohammadi-Nasrabadi

**Affiliations:** 1 Microbiology and Food Hygiene Department, Faculty of Veterinary Medicine, Lorestan University, PO Box: 44316-68151, Khorramabad, Khorramabad, Lorestan, 6815144316 Iran; 2 Nutritional Health Research Center, School of Health and Nutrition, Lorestan University of Medical Sciences, Khorramabad, Iran; 3 Food and Nutrition Policy and Planning Research Department, Faculty of Nutrition Sciences and Food Technology, National Nutrition and Food Technology Research Institute, Shahid Beheshti University of Medical Sciences, Tehran, Iran

**Keywords:** Monte carlo simulation, Heavy metals, Small ruminant, Risk assessment, Iran, Diseases, Environmental sciences, Medical research, Risk factors

## Abstract

Potentially toxic metal contamination in edible animal tissues is a major concern for food safety and public health. This study investigated the concentrations of essential and potentially toxic metals in the meat and liver of eighty small ruminants in Lorestan, Iran and assessed the risks associated with their consumption. Essential (Co, Se, Cu, Fe, Mg, Mn, and Zn) and potentially toxic (Ni, Cd, and Pb) metal concentrations were measured using ICP-OES. The sources and distribution patterns of essential and toxic trace metals were examined using multivariate statistical analyses including the Pearson correlation coefficient (PCC), principal component analysis (PCA) and hierarchical cluster analysis (HCA). Human health risk was evaluated using the lifetime cancer risk, the hazard ratio and the hazard index, and uncertainty in these parameters was quantified through Monte Carlo simulation. Most metals were present at concentrations consistent with international standards, whereas Zn with values from 75.33 to 132.5 mg/kg, Ni with values from 0.76 to 1.2 mg /kg and Pb with values from 0.12 to 0.65 mg/kg exceeded the limits recommended by FAO/WHO. The hazard index was less than one, indicating a low probability of non-carcinogenic effects. However, the lifetime cancer risk suggested a moderate carcinogenic risk associated with nickel in meat. Multivariate analysis showed that manganese, iron, copper and cadmium were primarily associated with natural terrestrial sources, while Ni, Pb and Zn were linked to anthropogenic inputs. These findings highlight the need to assess grazing environments and water sources, improve hygienic practices during slaughter and establish maximum permissible levels for trace elements to reinforce food safety monitoring.

**Supplementary Information:**

The online version contains supplementary material available at 10.1038/s41598-025-33838-2.

## Introduction

 Meat and its byproducts represent an important component of the human diet, providing high-quality proteins and a wide range of essential nutrients. Red meat, in particular, is recognized for its substantial content of trace minerals such as zinc (Zn), calcium (Ca), selenium (Se), and iron (Fe), along with several vitamins vital for physiological functions^1^. However, despite these nutritional advantages, red meat can also serve as a reservoir for potentially harmful substances, including potentially toxic metal, which may contribute to adverse health outcomes when present at elevated concentrations^2^. In small ruminants, such as sheep and goats raised under extensive grazing systems, the accumulation of trace elements in edible tissues and organs is strongly influenced by the mineral composition of their forage and environment. Depending on their concentration and biological role, these elements can exert beneficial, neutral, or detrimental effects on animal performance and overall health^3^.

Heavy metals can be classified into two categories: essential and non-essential, based on their functions in biological systems. Essential heavy metals, including manganese (Mn), Fe, copper (Cu), and Zn, play crucial roles for living organisms and are needed in trace amounts within the body. In contrast, heavy metals like cadmium (Cd), lead (Pb), and mercury (Hg) are toxic and are regarded as biologically non-essential^4^.

Heavy metals pose significant environmental risks to living organisms, depending on the dose and duration of exposure. Key toxic metals of concern include chromium (Cr), nickel (Ni), Cu, Zn, Cd, Pb, Hg, and arsenic (As). Rapid urbanization and industrialization have increased the atmospheric burden of these metals, adversely affecting human health and ecosystem integrity. Contamination arises from both anthropogenic and natural sources, and these metals can re-enter soil and water systems through various biogeochemical pathways^5^.

The average levels of metals in Iranian agricultural soils follow the following order: Zn > Cr > Ni > Cu > Pb > As > Cd ^6^. By comparing the HM levels in agricultural soils of the study areas with the recommended soil values ​​provided by DOE^7^ and global soil standards (50 mg/kg for Ni and 1 mg/kg for Cd), the average levels of Ni (73.19 mg/kg) and Cd (1.02 mg/kg) were 1.4 and 1.02 times higher than the corresponding levels in the Iranian soil standard for agricultural soils, respectively^6^. Comparing Cd levels (0.21–1.03 mg/kg; mean 0.73 mg/kg) in Pakistan^8^ with those in Iran also shows that this region has Cd levels in agricultural soils exceeding the WHO guidelines (0.003 mg/kg), making the soil unsuitable for agricultural use.

Additionally, natural water bodies face significant environmental challenges from industrial discharges, mining operations, agricultural runoff, and poor waste disposal. Research indicates that the levels of Zn, manganese, Cu, As, Cd, Cr, and Ni in western Iran^9^ exceed the WHO and EPA limits. Similar issues are found in Pakistan, where groundwater levels of heavy metals also surpass WHO standards^10^. Cd, Zn, and Ni are predominantly of human origin, whereas lead has both anthropogenic and natural sources, and copper and manganese are primarily derived from natural processes^11^.

Over time, heavy metals accumulate from water and vegetation and enter the food chain. Biomagnification then elevates their concentrations at higher trophic levels, creating serious health risks for humans^5^. High levels of heavy metal contamination have been found in meat, organs (such as liver and kidney), and eggs (particularly in domestic chickens) in regions like Iran, India, Pakistan, and Bangladesh. This contamination is attributed to environmental factors, including industrial waste, contaminated animal feed, and certain agricultural practices^12–16^. In heavily polluted urban areas, additional sources such as industrial emissions contribute to the problem^14^.

Regular monitoring of toxic and essential trace elements in livestock raised under extensive production systems is vital for evaluating their concentrations in meat and edible offal. This monitoring ensures that residue levels remain within the maximum limits established for human consumption and helps verify adequate mineral nutrition, since suboptimal tissue trace element levels may indicate deficiencies or imbalances that reduce productivity^17^. Additionally, trace element residues found in animal tissues act as valuable signals of environmental pollution stemming from both natural and anthropogenic sources, and can help inform strategies aimed at reducing the environmental effects of livestock farming^17^. Several studies have also documented asymptomatic or subclinical accumulation of heavy metals in animals, where elevated tissue concentrations occur without overt clinical signs and are detectable only through biochemical or pathological analyses^18,19^.

Accurate determination of trace metals is analytically challenging because their concentrations are often very low, which requires the use of highly sensitive and precise analytical techniques. Flame atomic absorption spectrometry (FAAS) and electrothermal atomic absorption spectrometry (ET-AAS) are commonly applied for measuring metals at parts per million to parts per billion levels. For ultra trace determinations, inductively coupled plasma optical emission spectrometry (ICP-OES) provide higher sensitivity and allow simultaneous measurement of multiple elements. Among these techniques, ICP-OES is widely used in food and meat analysis because it offers accurate, reproducible and multi element quantification of essential and toxic metals^20,21^. Understanding the occurrence and distribution of these metals is especially important when applying chemometric tools such as the Pearson correlation coefficient (PCC), principal component analysis (PCA), and hierarchical cluster analysis (HCA) to interpret contamination patterns^22,23^. Therefore, the present study aims to provide comprehensive insight into meat quality and contamination by assessing the levels of essential and heavy metals.

Consequently, this study focused on determining the average concentration of Cd, Ni, Co, Se, Pb, Cu, Fe, Mg, Mn, and Zn in the muscle and liver of goats and sheep that were slaughtered in southwest Iran. Moreover, the target hazard quotient (THQ), hazard index (HI), and Lifetime Cancer Risk (ILCR) of the muscle and liver were assessed. Therefore, this study is the first to assess the health risks to consumers posed by toxic metals/essential trace elements in muscle and liver of slaughtered small ruminants through Monte Carlo simulations. Furthermore, the distribution patterns and potential sources of toxic and trace elements in the samples were assessed using multivariate statistical techniques, including PCC, PCA, and HCA.

## Materials and methods

### Sampling

**Fig. 1:**
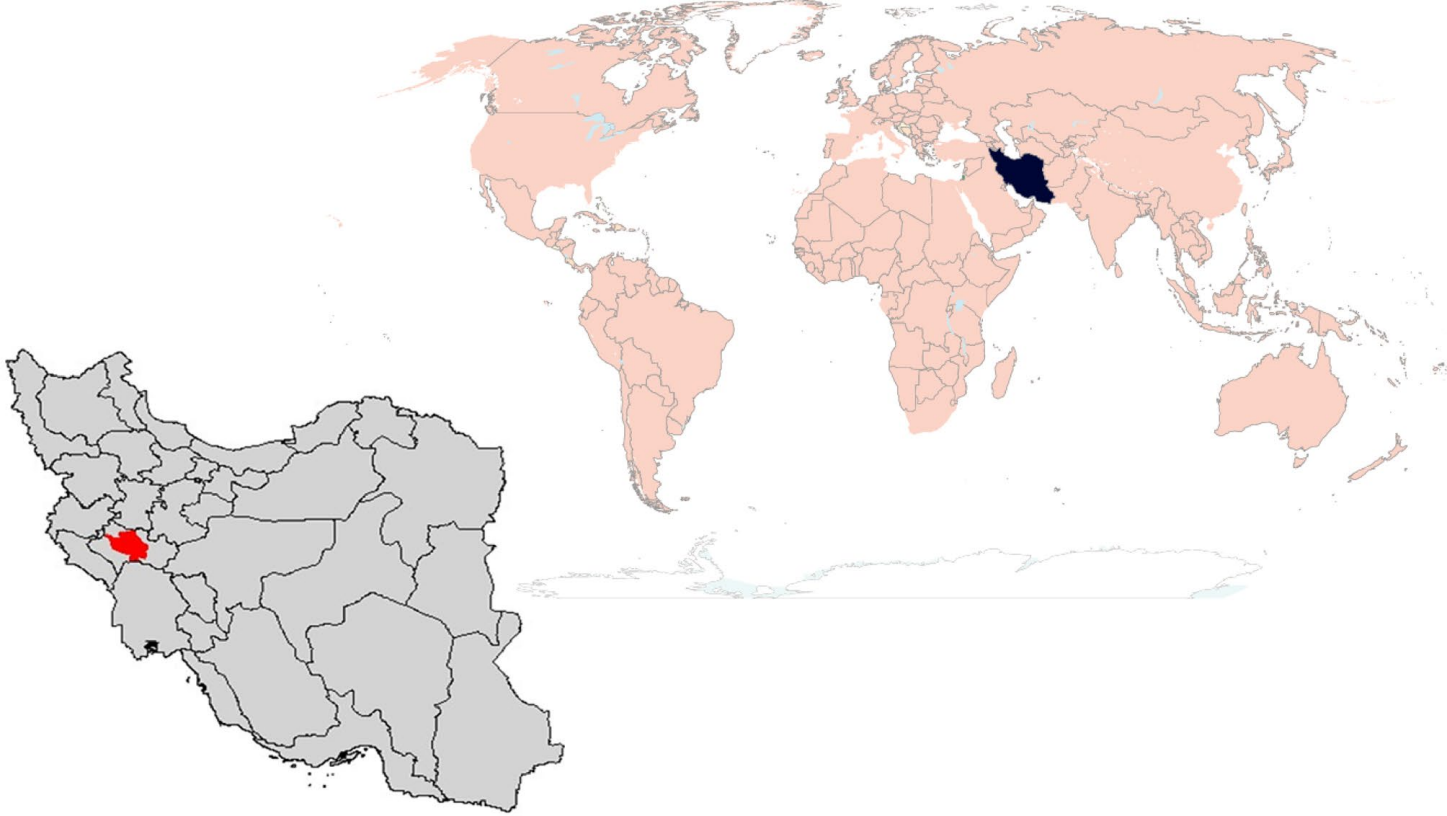
Geographic location of the sampling site used in the study.

The study was conducted in Lorestan Province (33°34′N, 48°24′E) in western Iran, which has an estimated population of approximately 1,760,649 residents. The investigation focused on Khorramabad, the provincial capital, where the Golshan Slaughterhouse, the only livestock slaughterhouse in the city, provides meat for the local population (Fig. 1). From October to December 2023, weekly visits were made to the goat and sheep slaughtering sections of the selected slaughterhouse. During each visit, approximately five goat carcasses and five sheep carcasses were collected using a systematic random sampling approach, selecting one carcass for every five.

A total of 80 samples weighing approximately 50 g each of breast muscle (meat) and liver were obtained from healthy sheep (20 rams and 20 ewes) and goats (20 males and 20 females). The evaluation of animal health was conducted through a comprehensive examination by a licensed veterinarian. The animals selected for sampling were aged between 2 and 4 years, with age determination achieved through an assessment of their dental condition during the examination. The samples were collected in PET (polyethylene terephthalate) containers and transferred to the food hygiene laboratory of the Faculty of Veterinary Medicine of Lorestan University. The samples were appropriately labeled and stored at −18 °C until analysis.

### Samples Preparation

All reagents used were of analytical-grade purity, including 30% hydrogen peroxide, 70% perchloric acid, and 65% nitric acid (Merck, Germany). Prior to sample preparation, all laboratory equipment was soaked in a soap solution for at least 2 h, thoroughly washed with tap water, immersed in 10% nitric acid overnight, and finally rinsed with deionized distilled water. Liver and muscle samples were oven-dried at 60 °C for 24 h. The dried tissues were then ground using a mortar and pestle. Subsequently, 0.5 g of each sample (muscle and liver) was subjected to acid digestion in a high-pressure digestion vessel (Model 4746, Parr Instrument Co., Moline, IL). Each sample was treated with 5 mL of 65% HNO₃, 2 mL of 37% HCl, and 1 mL of 30% H₂O₂ in a beaker. The digestion vessels were heated to 140 °C for 130 min and then allowed to cool to room temperature. Digestion was continued until the samples were completely dissolved, with no visible particulate matter remaining^24^. The digests were filtered through 0.45-µm membrane filters (Whatman, Merck, Germany), diluted to a final volume of 25 mL with chromatography-grade water, and stored in Falcon tubes until analysis.

The concentrations of the heavy metals were determined by means of an Inductively Coupled Plasma-Optical Emission Spectrophotometer (ICP-OES, Agilent 5110, USA). All parameters for ICP-OES were applied as indicated in Table 1.

**Table 1:** ICP-OES instrumental and operational conditions.

Parameters	Value/type
GENESIS-EOP	
RF power/W	1400
Readings/replicate	2
Coolant flow rate/L min-1	12
Auxiliary flow rate/L min-1	1
Nebulizer flow rate/L min-1	1
Nebulizer	Modified Lichte Spectro
Optic temperature/°C	29.65
Element/λ/nm	Cd 214.438, Pb 220.353, Ni 221.648, Fe 238.204, Zn 213.856, Cu 324.754, Co 228.616, Mg 285.213, Mn 257.611, Se 196.090,

Instrument calibration was conducted using multi-element standard solutions corresponding to the target metals, which were employed to construct standard calibration curves prior to elemental analysis. Individual standard solutions were prepared from certified 1000 mg/L stock solutions. For the calibration of metals multi-element standards were prepared through serial dilution from intermediate 100 ppm solutions. The calibration curves were generated by introducing both the standard solutions and a blank into the ICP-OES instrument. Metal concentrations in the samples expressed as mg/kg. Blank and reference solutions underwent identical preparation and analytical procedures as the samples to ensure consistency and accuracy.

### Quality control

Standard solutions of the metals were created using the standard stock solution (NIST- SRM 1577b bovine liver) at three varying concentrations: 100, 500, and 1000 µg.kg⁻¹. The dilution process was carried out using distilled water. A standard curve was generated by injecting both standards and blank solutions into the ICP-OES^25^.

In this study, the limit of detection (LOD) for the samples, expressed in ppb, were found to be 0.942 for Pb, 0.160 for Co, 0.904 for Cu, 6.638 for Se, 0.749 for Ni, 0.127 for Cd, 0.225 for Fe, 1.0007 for Mg, 0.036 for Mn, and 0.173 for Zn. For reliable instrument performance, calibration values should exceed 0.99. Additionally, spiked samples were prepared. A recovery study was performed by adding varying concentrations of a multi-element standard solution to the samples under investigation and then re-measuring them. All experiments were carried out in triplicate. To ensure instrument reliability, after every 10 samples, a blank and a known standard were processed (relative standard deviation, RSD ≤ 3%). Acceptable recovery rates for the measured metals ranged from 90.4% to 103%^26^.

### Statistical analysis

After gathering the required data, the analysis was conducted using the SPSS software (version 27, SPSS Inc., Chicago, IL, USA). Categorical variables were summarized using mean and standard deviation. The normality of the data was assessed using the Kolmogorov-Smirnov test, and one-way ANOVA followed by Duncan’s test was employed to detect significant differences in sex and species between meat and liver samples. Statistical significance was considered for P-values below 0.05.

### Health risk assessment

The present study aimed to understand the level of exposure to toxic elements from consuming meat and its products. Although some concentrations found in the study are not immediately toxic, they could be a concern for long-term consumption. Specifically, Cd, Ni, Co, Se, Pb, Cu, Fe, Mg, Mn, and Zn in sheep meat and offal were of interest^27^.

### Carcinogenic risk

Lifetime Cancer Risk (ILCR) is the probability that an individual will develop cancer during his lifetime due to exposure to carcinogens. The following equation is used to evaluate the risk of carcinogenesis:

$${\rm ILCR = EDI \times CSF}$$

ILCR: Lifetime Cancer Risk

CSF: Cancer slope factor.

Estimated Daily Intake (EDI) is the estimated daily intake (mg/kg/day) calculated using the following equation:

$${\rm EDI =} \:\frac{\mathrm{I}\mathrm{R}\:\times\:\:\mathrm{C}}{\mathrm{B}\mathrm{W}}$$

IR: Ingestion Rate (2 g per day for liver^28^ and 3.14 kg per year of meat^29^.

C: metal concentration in the sample (mg/kg).

BW: average body weight (70 kg for adults).

In this investigation, the ILCR associated with exposure to Pb, Cd, and Ni was assessed, with values recorded at 0.0085, 0.380, and 0.84, respectively. These estimations were derived from existing toxicological data pertinent to the cancer slope factor (CSF). The information for Co, Se, Cu, Fe, Mg, Mn, and Zn in the CSF is currently inaccessible, and as a result, their carcinogenic risk cannot be determined^30^. The USEPA states that a risk of less than 1 × 10^− 6^ can be considered negligible, while a risk greater than 1 × 10^− 4^ is likely hazardous to human health. A risk within the permissible risk range of 10^−4^−10^−6^ is considered acceptable or tolerable^31^.

### Non-carcinogenic risk assessment

Hazard quotients (HQ) were used to calculate the non-carcinogenic health, which refers to the ratio of exposure to heavy metals to the reference dose (RfD). The RfD in mg/kg/day depends on the specific pollutant. Its oral values for Cd, Ni, Co, Se, Pb, Cu, Fe, Mn, and Zn are 0.001, 0.02, 0.03, 0.005, 0.004, 0.04, 0.7, 0.14, and 0.3 mg/kg/day, respectively^32^. If its value is less than one, there is no non-carcinogenic risk; however, if its value is greater than one, the non-carcinogenic risk is unacceptable, and the possibility of a negative impact on human health or the need for additional investigation^31^.

$$\:HQ=\frac{EF\times\:ED\times\:IR\:\times\:C}{RfD\times\:BW\times\:\:AT\:}$$

EF: Exposure frequency (365 days in a year).

ED: Duration of human exposure (70 years).

IR: Ingestion rate (2 g per day for liver and 3.14 kg per year of meat).

C: Metal concentration in the sample (mg/kg).

BW: Average body weight (70 kg for adults).

AT: Averaging time of human exposure (25550 days)^33^.

Exposure to several pollutants can lead to combined and interactive impacts. To assess the potential risk linked to exposure to multiple metals, the hazard index (HI) was utilized. The HI was determined by adding together the HQ values:

$$\:HI={\sum\:}_{1}^{i}{HQ}_{s}$$

### Monte Carlo simulation and uncertainty analysis

The health risks associated with metal exposure from the consumption of meat and offal were assessed using a Monte Carlo simulation with 10,000 iterations at a 95% confidence level, performed in Crystal Ball (Oracle, USA)^23^. Probability distributions such as Beta, Log-normal, and Weibull were selected for the input data based on goodness-of-fit criteria and corresponding p-values. Health risk thresholds were evaluated at both the mean and the 95th percentile values of ILCR, HQ, and HI obtained from the simulation results.

Additionally, OriginPro 2025 was used to conduct statistical analyses to explore the interrelationships among elements. The dataset was thoroughly analyzed using several multivariate statistical methods. These included Pearson correlation coefficients (PCC) to evaluate pairwise associations, principal component analysis (PCA) to detect major patterns and potential sources, and hierarchical cluster analysis (HCA) to categorize elements with comparable characteristics or origins.

## Results and discussion

### Toxic/trace element concentrations in small ruminants

Monitoring the levels of toxic and trace elements in livestock is essential, particularly in extensive farming operations, to gain insights into the nutritional well-being of animals, the nutritional quality of meat, and the presence of potentially harmful element residues in meat products. Hence, it is essential to assess the levels of chemical pollutants in small ruminant meat and liver, given its high consumption in this city, to evaluate potential health risks. The results are presented by mean ± SD, and MRL values of Cd, Ni, Co, Se, Pb, Cu, Fe, Mg, Mn, and Zn in meat and liver in Tables 2 and 3.

**Table 2:** The mean ± SD of toxic elements in the muscle and liver of small ruminants (mg/kg) compare with MRL.

Toxic Elements (Mean ± SD)							
Samples (*N* = 80)		Cd		Pb		Ni	
		Muscle	Liver	Muscle	Liver	Muscle	Liver
Sheep	Ewe (*n* = 20)	ND	0.15 ± 0.01^a^	ND	0.12 ± 0.02 ^a^	0.83 ± 0.07^a^	0.79 ± 0.06 ^a^
	Ram (*n* = 20)	ND	0.12 ± 0.01^b^	ND	0.57 ± 0.02 ^b^	0.84 ± 0.06 ^a^	0.84 ± 0.07^a^
Goat	Female (*n* = 20)	ND	0.081 ± 0.01^c^	0.41 ± 0.04 ^c^	0.65 ± 0.02 ^d^	1.2 ± 0.27 ^b^	0.83 ± 0.05 ^a^
	Male (*n* = 20)	ND	0.14 ± 0.01^d^	0.23 ± 0.01 ^e^	0.29 ± 0.01 ^f^	0.76 ± 0.02 ^a^	0.78 ± 0.03 ^a^
MRL		0.05^*^	0.50^*^	0.10^**^	0.50^**^	0.5^***^	0.5^***^

ND, not detected ^a, b, c, e, d, f, g,^ Significant difference between the muscle and liver in sheep and goat (*p* < 0.05) Duncan’s test in the same line. MRL (maximum residue level): EC (European Commission) ^*^, Codex Alimentarius^**^, WHO^***^.

**Table 3:** The mean ± SD of essential trace elements in the muscle and liver of small ruminants (mg/kg) compare with MRL.

		Essential Trace Elements (Mean ± SD)													
Samples (*N* = 80)		Co		Se		Cu		Fe		Mg		Mn		Zn	
		Muscle	Liver	Muscle	Liver	Muscle	Liver	Muscle	Liver	Muscle	Liver	Muscle	Liver	Muscle	Liver
Sheep	Ewe (*n* = 20)	ND	0.070 ± 0.03^a^	2.5 ± 0.24^a^	2.7 ± 0.22^a^	9.6 ± 0.029^a^	30 ± 0.093^b^	112.7 ± 3.84^a^	214.4 ± 2.80^b^	717.3 ± 36.60^a^	535.7 ± 13.21^b^	0.71 ± 0.14^a^	6.6 ± 0.28^b^	102.9 ± 5.10 ^a^	86.26 ± 2.88^b^
	Ram (*n* = 20)	ND	0.084 ± 0.03^a^	2.0 ± 0.07^b^	2.0 ± 0.09^b^	9.4 ± 0.33^a^	32 ± 0.63^c^	113.3 ± 1.79^a^	233.0 ± 1.45^c^	865.7 ± 22.30^c^	546.6 ± 17.24^b^	1.8 ± 0.10 ^c^	8.1 ± 0.10^d^	106.1 ± 2.30 ^a^	132.5 ± 0.87^c^
Goat	Female (*n* = 20)	ND	0.22 ± 0.04^b^	2.0 ± 0.18^c^	2.2 ± 0.14^d^	9.6 ± 0.27^a^	34 ± 0.64^d^	118.2 ± 1.72^d^	225.7 ± 2.63^e^	633.3 ± 21.16^d^	614.4 ± 4.35^d^	1.0 ± 0.17 ^a^	6.0 ± 0.39^e^	113.7 ± 2.71 ^d^	96.53 ± 0.92^e^
	Male (*n* = 20)	ND	0.31 ± 0.09^c^	2.0 ± 0.06^c^	2.2 ± 0.09^d^	9.4 ± 0.28^a^	36 ± 0.62^f^	115.3 ± 1.11^d^	226.7 ± 0.98^e^	356.8 ± 17.97^e^	458.6 ± 17.49^f^	0.98 ± 0.10^a^	5.1 ± 0.41^f^	113.0 ± 1.04 ^d^	75.33 ± 0.46^f^
MRL		-	-	-	-	40^*^	40^*^	-	-	-	-	3.99^**^	0.5^**^	50^**^	80^**^

ND, not detected ^a, b, c, e, d, f, g,^ Significant difference between the muscle and liver in sheep and goat (*p* < 0.05) Duncan’s test in the same line. MRL (maximum residue level): Codex Alimentarius^*^ and FAO/WHO^**^.

### Toxic metal

#### Cadmium (Cd)

Cd in goat and sheep meat samples was lower than the detection limit. Cd concentrations in liver samples were 0.15 mg/kg and 0.12 mg/kg for ewes and rams and 0.081 mg/kg and 0.14 mg/kg for male and female goats, respectively (Table 2). The mean concentration of Cd in the livers of goats and sheep exhibited significant variations based on both gender and species (*p* < 0.001). The average concentration of Cd found in liver samples was significantly below the MRL (0.5 mg/kg)^34^, demonstrating the safety and compliance of the findings in this study. Additionally, male goats had significantly higher amounts of Cd than female goats (*p* < 0.002). Our results also showed that the mean of Cd in ewe liver was significantly higher than that of ram (*p* < 0.001).

The study from Sanandaj, Iran, that investigated the levels of various heavy metals in sheep showed the mean cadmium concentrations in sheep were 2.80 ± 1.98 mg/kg^35^, which is in agreement with the present study. In a study conducted in southeastern Nigeria, Cd level in goat liver tissue was found to be 0.02 mg/kg, which is lower than the value reported in our current study. However, unlike our findings, which showed that cadmium was not detected in goat muscle tissue, their study reported a concentration of 0.02 mg/kg in that tissue^36^. Furthermore, the Cd concentrations in the muscle and liver of lambs and sheep in the Czech Republic (during 2001–2022) were 0.004 ± 0.001 and 0.319 ± 0.047 mg/kg, respectively^37^. Dietary cadmium exposure significantly increased cadmium accumulation and upregulated the mRNA expression of metal transporter genes (divalent metal-ion transporter-1, Zrt-/Irt-like protein, and ferroportin-1) in the liver and kidney, but not in muscle, indicating tissue-specific differences in Cd deposition among the liver, kidney, and muscle of goats^38^.

The variation in levels of Cd is probably due to factors such as differing environmental pollution, sources of feed, the age of the animals, and the amounts of Cd present in animal feed, which can affect the Cd levels in animal tissues. Cd, recognized as an environmental pollutant, is easily absorbed by plants from the soil and subsequently transferred to animals through the plants they eat^39^. Animals grazing in natural pastures are exposed to higher levels of Cd ^37^. In this province, goats and sheep are primarily raised in extensive grazing systems. As a result, they may accumulate higher amounts of Cd compared to cattle, making them good indicators of cadmium pollution levels in a particular area.

### Lead (Pb)

The average Pb levels found in the livers of ewes were notably higher (0.12 mg/kg) than those in rams (0.57 mg/kg). Furthermore, Pb concentrations in sheep meat samples were lower than the detection limit. In goats, Pb concentrations were higher in females than in males, with statistically significant differences observed based on sex and species in both meat and liver tissues (*p* < 0.0001). According to Table 2, Pb concentrations in male sheep and female goats were above the MRL (0.500 mg/kg in liver, 0.1 mg/kg in meat)^34^.

Results of Bazargani-Gilani, et al. ^40^ research in Hamedan, Iran, showed that the level of Pb in the livers of male sheep (40.44 mg/kg) was higher than that in female sheep (9.25 mg/kg); it was revealed that the Pb level in the livers of male sheep (40.44 mg/kg) exceeded that of female sheep (9.25 mg/kg). Conversely, for goats, the Pb concentration in females (1.73 mg/kg) was greater than in males (1.68 mg/kg)^40^, which aligns with the current research. However, the levels are significantly higher than those observed in the present study. Numerous studies have reported Pb contamination of soil and various products in Iran^16,41^. The average Pb concentration in Iranian agricultural soils is approximately 1.5 times higher than the global mean. Major sources contributing to Pb contamination include vehicular emissions, irrigation with municipal wastewater, agricultural practices and machinery use, mining and smelting activities, operations of oil refineries and petrochemical industries, as well as naturally occurring terrestrial inputs^6^. Upon ingestion, Pb accumulates in biological tissues, leading to increased toxicity due to its persistent nature and adverse chemical properties^18^.

A systematic study found high Pb contamination levels in red meat, particularly in Asia (1020.15 µg/kg) and Africa (965.73 µg/kg), while the Americas and Europe had lower levels^13^. This contamination is linked to pollution from petrochemical industries in these productive regions^42^. China and India, as major meat producers, contribute to this issue, with research showing Pb concentrations in Chinese meat samples reaching 2884 µg/kg^43,44^. Additionally, many animal feed samples in these areas contained excessive Pb, likely from contaminated soil consumed by animals, further increasing Pb levels in the food chain.

### Nickel (Ni)

The average concentration of Ni in the liver and meat of small ruminants studied ranged from 0.76 to 1.2 mg/kg, which in all samples was more than 0.5 mg/kg recommended by WHO^45^. The values obtained from this study exceed those reported by other researchers. One study recorded the concentration of Ni in goat liver as 0.188 ± 0.001 ppm and in goat meat as 0.063 ± 0.003 ppm^12^. In another investigation involving goats, the concentrations of Ni in liver tissues were noted as 0.19 ppm^46^. In a study conducted in Kurdistan, Iran, the nickel level in sheep was determined to be 0.54 ± 0.57 (Raeeszadeh, Gravandi, & Akbari, 2022). Furthermore, research conducted in India found the mean concentrations of Ni in goat liver and muscle to be 0.350 ± 0.050 and 0.552 ± 0.073 ppm, respectively^35^. However, in India, the mean Ni concentration in goat liver and muscle was found to be 0.350 ± 0.050 and 0.552 ± 0.073 ppm, respectively^47^. The main route of environmental Ni exposure is through soil transport^48^. The mean concentration of Ni in Iranian agricultural soils is approximately 4.3 times higher than the global average and exceeds the maximum permissible limit of 50 mg/kg by 1.5 times. This elevated level of Ni raises substantial environmental concerns, particularly regarding its potential impact on soil quality, plant uptake, and ecosystem health^6^. Variations in dietary intake among species—attributable to differences in livestock farming systems—can account for observed disparities in Ni exposure^35^.

Ni is not considered a bioaccumulative element within the food chain; nonetheless, exposure to Ni can result in several adverse health effects. These effects may include respiratory dysfunction, oxidative stress, skin allergies manifesting as rashes, or other dermatological issues. Further health concerns associated with Ni include hepatotoxicity, immunotoxicity, and the risk of developing cancer over time^49^. Given the adverse effects of Ni, this may be a potential health risk, particularly for adults.

### Essential trace metals

Trace elements are crucial for maintaining good health, and meat and meat products are significant sources of these elements in the human diet. While there have been reports on normal or sufficient trace element levels in meat and edible organs, the EU has not established minimum and maximum limits for essential trace elements in meat and meat products.

### Cobalt (Co)

As shown in Table 3, average Co levels in the liver are greatest in male goats (0.31 mg/kg), followed by female goats (0.22 mg/kg), rams (0.084 mg/kg), and ewes (0.07 mg/kg). There were significant differences in Co concentrations related to species and sex in the goat livers (*p* < 0.05). The data indicates that levels were notably higher in goats than in sheep and significantly higher in males than in females. The Co concentrations noted in this research were comparable to those observed in the liver, kidney, and meat of cattle, sheep, and goats from the gold-mining regions of Zamfara State, Nigeria, with values ranging from 0.00 to 2.71 µg/g ^50^. The Co content observed in this study is lower than that reported in goat and mutton meat from Iran^35^, Pakistan^51^, Taiwan, and Sweden, which were 0.043, 0.033, and 0.001 µg/g, respectively^52^. Furthermore, comparison with studies conducted in Nigeria indicates that Co levels in the meat and liver tissues of goats and sheep were also lower^50,53^. To date, a definitive tolerance limit for Co has yet to be established; however, various studies have identified adverse effects associated with both short-term and long-term exposure durations. Co is emitted into the atmosphere due to natural and human activities. It is important for vitamin B_12_, but consuming too much can negatively impact the endocrine, nervous, and cardiovascular systems^54^. Furthermore, recent findings suggest that cobalt poisoning may lead to alterations in vision, peripheral nerve damage, loss of hearing, and decreased thyroid function, as well as the development of respiratory tumors in the affected area^55^.

### Selenium (Se)

Sex-specific variations in Se levels were significant in sheep (*p* < 0.001), whereas no notable differences were detected in liver and meat compositions. The amount of Se in goats was not significantly different between males and females (*p* > 0.99); however, mean Se was higher in the liver. The mean Se levels in sheep and goat liver were 2.292 mg/kg, while in their meat, it was 2.113 mg/kg. In contrast to the findings of the present study, an Australian study on sheep reported mean Se concentrations of 0.09 mg/kg in muscle and 0.31 mg/kg in liver^27^. On the other hand, the mean Se levels in sheep muscle and liver tissues were 0.09–0.4 and 0.5–0.25 mg/kg, respectively, which are lower than the Se levels found in the present study^17^. The concentration of tissue Se in the samples was high and may reflect the presence of known areas with high environmental levels of Se in some pastures of this province, along with the presence of some low-accumulated plants in the pasture^27^. In this research, the level of Se found in the liver was greater than that in the meat, likely due to the significant increase in liver Se levels when animals are given selenium-enriched dietary supplements. For instance, lambs that were given selenium-enriched yeast exhibited liver selenium concentrations of 22.64 mg selenium/kg dry matter, whereas this level was lower in muscle tissues (Juniper et al., 2008). On the other hand, a lack of Se can result in decreased liver selenium levels, negatively impacting overall health and metabolic functions^56^. Se levels in lambs can be significantly impacted by the accumulation of toxic metals within the liver, particularly in instances where the livestock are grazing on pastures that have been treated with sewage sludge^57^.

The differences in Se levels among states indicate the existing environmental concentrations. The concentration of tissue Se in the samples was high and may reflect the presence of known areas with high environmental levels of Se in some pastures of this province, along with the presence of some low-accumulated plants in the pasture^27^.

Se is an essential element for the growth and fertility of livestock; however, the range between deficiency and toxicity is very narrow, requiring careful management of nutrition and environmental resources. The critical level of Se in livestock for deficiency in cattle is less than 0.10 ppm, and in sheep, it is less than 0.03 ppm, and for toxicity, it is reported to be 1 to 5 ppm^58^. The Se measured in this study is within this range for both goats and sheep.

Soil, particularly heavy soils with an appropriate pH, along with water, are the primary sources of Se for plants. Plants that grow in selenium-rich soils may accumulate Se in their tissues^58^. Some selenium-accumulating species, such as milk vetch and poison vetch, are typically avoided by livestock due to their unpleasant odor; however, they may be consumed during periods of forage scarcity^59^. An accumulation of more than 5 ppm of Se in plants can pose risks to livestock and result in selenosis (selenium poisoning), which manifests in three forms: acute, subacute, and chronic^58^.

### Copper (Cu)

Table 3 reveals critical data regarding the Cu levels found in the liver and meat of sheep and goats. Impressively, the liver of the male goat boasts the highest Cu concentration at 36 mg/kg, highlighting its superior nutritional value, while the ram and goat meat show the lowest level at 9.4 mg/kg. Notably, all Cu levels in these samples are below the safe threshold of 200 ppm^60^ and 40 ppm^61^, ensuring consumer safety. It’s important to emphasize that Cu concentrations in liver samples are significantly greater than those in meat across all species (*p* < 0.001). Previous research conducted in Bangladesh indicated that the Cu concentrations in goat meat and liver were reported at levels of 94.25 ± 0.28 mg/kg and 473.99 ± 2.81 mg/kg, respectively. These findings are significantly higher compared to the results observed in the current study^62^. Variations in heavy metal levels can be attributed to the age, diet, bioaccumulation mechanisms, and sex of the animal, since these elements influence the buildup of heavy metals in their tissues^63,64^.

Furthermore, previous studies consistently confirm that the liver is a rich source of Cu, ranking higher than the kidney, meat, and blood, underscoring the liver’s vital role in nutrition^40^. Chronic Cu poisoning can result from prolonged exposure to high Cu levels found in Cu-based pesticides, commercial feeds meant for other animals, treated drinking water, fertilized pastures, and manure. Sheep are particularly vulnerable to toxic Cu accumulation due to their inability to excrete excess Cu, while goats have a lower capacity for Cu storage^65^. The concentrations of Cu in goat meat were below the marginal level of 10 mg/kg, with an average value of 9.4 mg/kg. This is significantly lower than the Cu levels found in goat meat worldwide. The difference is attributed to species-specific differences in Cu utilization between goats and sheep. Additionally, the grazing behaviors of sheep and goats differ, particularly in terms of their preferred feeding areas, types of plants, and parts of plants. Goats are adept at browsing and typically prefer a wider variety of shrubs, woody plants, weeds, and thorny bushes instead of grazing on grasses. Moreover, goats generally require lower mineral supplementation than sheep^65^.

#### Iron (Fe)

The livers of rams and male goats had the highest values (233 and 226.7 mg/kg) compared to those of ewes (214.4 mg/kg), with significant differences between species (*p* < 0.05). In the current study, Fe in all samples fell within the recommended tolerable levels by EU^66^. The typical concentration of Fe in food products generally falls within the range of 30 to 150 ppm. However, this range is notably elevated in organ meats such as the liver and kidney, where Fe levels can vary between 30 and 300 mg/kg ^39^. Consistent with the findings of the current study, Bazargani-Gilani, et al. ^40^ reported a significant discrepancy in Fe levels in the livers and kidneys of female sheep compared to male sheep, with a notably elevated Fe concentration. Moreover, the Fe levels in the livers of male goats were significantly higher than those found in female goats^40^. Since the liver and kidneys play a crucial role in eliminating toxic metals from the body, these two organs showed the highest levels of metal contamination in the animals analyzed^39^. The values obtained in the present study were significantly higher than those reported in studies from Nigeria^53^ and Uganda^67^, but lower than those from a study in Bangladesh^62^. Variability in Fe content within ruminant muscle and liver tissues is influenced by multiple interrelated factors, including geographic origin, production systems, animal age, specific muscle type, and liver composition^68,69^. Environmental conditions, such as exposure to sewage, can significantly affect hepatic Fe concentrations, particularly in bovine liver. Nutritional diet al.so plays a critical role; cattle raised on pasture generally exhibit higher Fe levels in muscle tissue compared to those fed concentrate-based diets. Additionally, muscles with higher oxidative capacity (aerobic muscles) tend to contain greater iron concentrations. While the liver contains a higher total iron content than muscle, it has a lower proportion of haem Fe, which is the more bioavailable form^68^. Variations in hepatic moisture and fat content, as well as differences in animal health and age, further contribute to the observed heterogeneity in Fe levels^69^. These findings underscore the importance of standardized methodologies for sampling and reporting to ensure accurate comparisons across studies.

### Magnesium (Mg)

The highest levels of Mg were observed in the meat of ewes and rams, ranging from 717.3 to 865.7 mg/kg, respectively. In contrast, the Mg levels in the livers of sheep and goats were found to be the lowest, at 535.7 and 546.6 mg/kg, respectively. Statistically significant differences (*p* < 0.001) were identified in Mg levels based on the sex and tissues of small ruminants (Table 3). The study conducted by Wang, et al. ^70^ identified notable variances in mineral concentrations between Alxa League goats and sheep, specifically highlighting Mg levels of 725 ± 171 mg/kg and 847 ± 160 mg/kg, respectively, as well as Fe content at 72 ± 18 mg/kg for goats and 95 ± 19 mg/kg for sheep, and Cu levels of 4.33 ± 2.26 mg/kg for goats compared to 5.79 ± 2.84 mg/kg for sheep, with statistical significance confirmed (*p* < 0.05)^70^. Moreover, the research underscores the impact of geographic location on the mineral composition of meat, emphasizing that animals rely on plant-based sources to obtain trace elements from the soil. As such, micronutrient deficiencies in forage can adversely affect livestock’s overall nutrition and health^71^.

#### Manganese (Mn)

Mn concentrations in the livers of rams were significantly higher than those in ewes, measuring 81 mg/kg in rams compared to 6.6 mg/kg in ewes, suggesting a potential influence of sex-based physiological differences. This aligns with the understanding that the liver serves as the primary organ for the storage and regulation of Mn levels^39^. Additionally, the findings corroborate previous research by ^40^, which reported even higher liver Mn concentrations. The mean Mn levels determined in the meat and liver tissues of goats in this study were higher than the values reported by Mushtaq, et al. ^51^and Soh, et al^72^. but lower than the values reported by Akan, et al. ^53^. Mn accumulation in liver tissues significantly exceeded the respective maximum safe limit (Table 3). Soil composition and distribution play significant roles in determining Mn levels, influenced by factors such as soil pH, plant species diversity, organic matter content, and the presence of silt and clay^73^. Furthermore, the use of Mn fertilizers can alter the Mn concentrations in crops^74^. The movement of Mn from the soil to plants is also influenced by various factors, including the concentrations of other cations (iron and aluminum), organic matter, cation exchange capacity, temperature, and biological activity (microbial and plant root exudative)^73^.

#### Zinc (Zn)

There was a significant difference in the Zn levels in the liver among the studied species (*p* < 0.05). As shown in Table 3, gender significantly affected the Zn concentration in liver. Rams had a liver Zn level of 132.5 mg/kg, significantly higher than the 86.26 mg/kg found in ewes. Furthermore, the Zn concentrations in meat, liver, and kidneys were determined to be 0.014, 1.05, and 0.44 mg/kg, respectively, in another study^27^. These results align with other studies, which demonstrate the influence of gender and species on Zn concentrations in the liver and meat of the examined animals^40^. The Zn content in all meat and liver samples surpassed the recommendations established by FAO/WHO^61^. Moreover, all Zn values in these samples fell below the acceptable threshold (< 150 ppm) set by the Australian and New Zealand Food Authority (ANZFA).

In Iran, Zn has entered agricultural soils predominantly through anthropogenic activities, including the application of chemical fertilizers, disposal of sewage, and the accumulation of waste from streetlights, batteries, and electrical appliances^6^. Environmental contamination can significantly influence the distribution, mobility, and bioavailability of trace elements such as Zn. In particular, heavy metal pollutants can interact with trace elements, modify their chemical behavior, and alter their uptake and accumulation in biological systems^39^. Supporting this, Zn concentrations in the kidneys of animals from unpolluted areas were reported to be higher than those from polluted regions, indicating possible disruption in Zn uptake or retention under polluted conditions^75^. Beyond environmental contamination, the inherent Zn content in agricultural soils can also influence Zn uptake by forage plants and, subsequently, by grazing livestock, thereby contributing to Zn accumulation in animal tissues. On average, Zn concentrations in Iranian agricultural soils are approximately 1.6 times higher than the global average. Certain regions, such as Hamedan in western Iran, exhibit Zn levels nearly twice the permissible threshold, posing potential ecological and health concerns^6^.

Research investigating the levels of trace elements in small ruminants has produced a spectrum of findings with respect to Zn content in muscle and liver tissues. For instance, a study by Khan, et al^76^. found that in goats, Zn concentrations in the liver and muscle were significantly elevated when compared to kidney tissues. Furthermore, Pannier, et al^77^. reported that the average Zn concentration in the longissimus muscle of lambs was 2.43 mg/100 g, which is recognized as a beneficial dietary source for the general population. It is noteworthy that Zn concentrations in the liver are closely associated with the levels of Cu and Zn present in a metallothionein-like protein fraction within the liver of calves and sheep, as demonstrated by^78^. Additionally, the concentrations of various minerals can be modulated by several factors, including the age at which the animal is slaughtered, which has a positive correlation with both iron and Zn levels in muscle tissue^77^. Collectively, these findings suggest that the distribution of Zn in the tissues of small ruminants may exhibit variability influenced by species-specific characteristics and other pertinent factors.

Significant variations have been noted in the findings of the reviewed studies, influenced by several factors such as the species of animals examined, the specific parts of the animals consumed, the geographical locations from which the samples were taken, and the dietary patterns of different population groups. Unsurprisingly, the highest concentrations of contaminants were detected in regions with environmental pollution from specific elements. It is well recognized that humans primarily encounter chemical pollutants, both organic and inorganic, through their diets. For populations not exposed occupationally, the intake of essential and non-essential trace elements largely depends on dietary consumption^79^.

#### Health risk assessment

The presence of toxic and essential trace elements in the meat and liver of small ruminants can present potential health risks for consumers. In Khorramabad, an evaluation was conducted to assess the risks associated with consuming meat and liver from slaughtered small ruminant carcasses. This assessment was based on the Estimated Daily Intake (EDI), Target Hazard Quotient (THQ), Hazard Index (HI), and Incremental Lifetime Cancer Risk (ILCR) methodologies.

### Non-carcinogenic risk

**Fig. 2:**
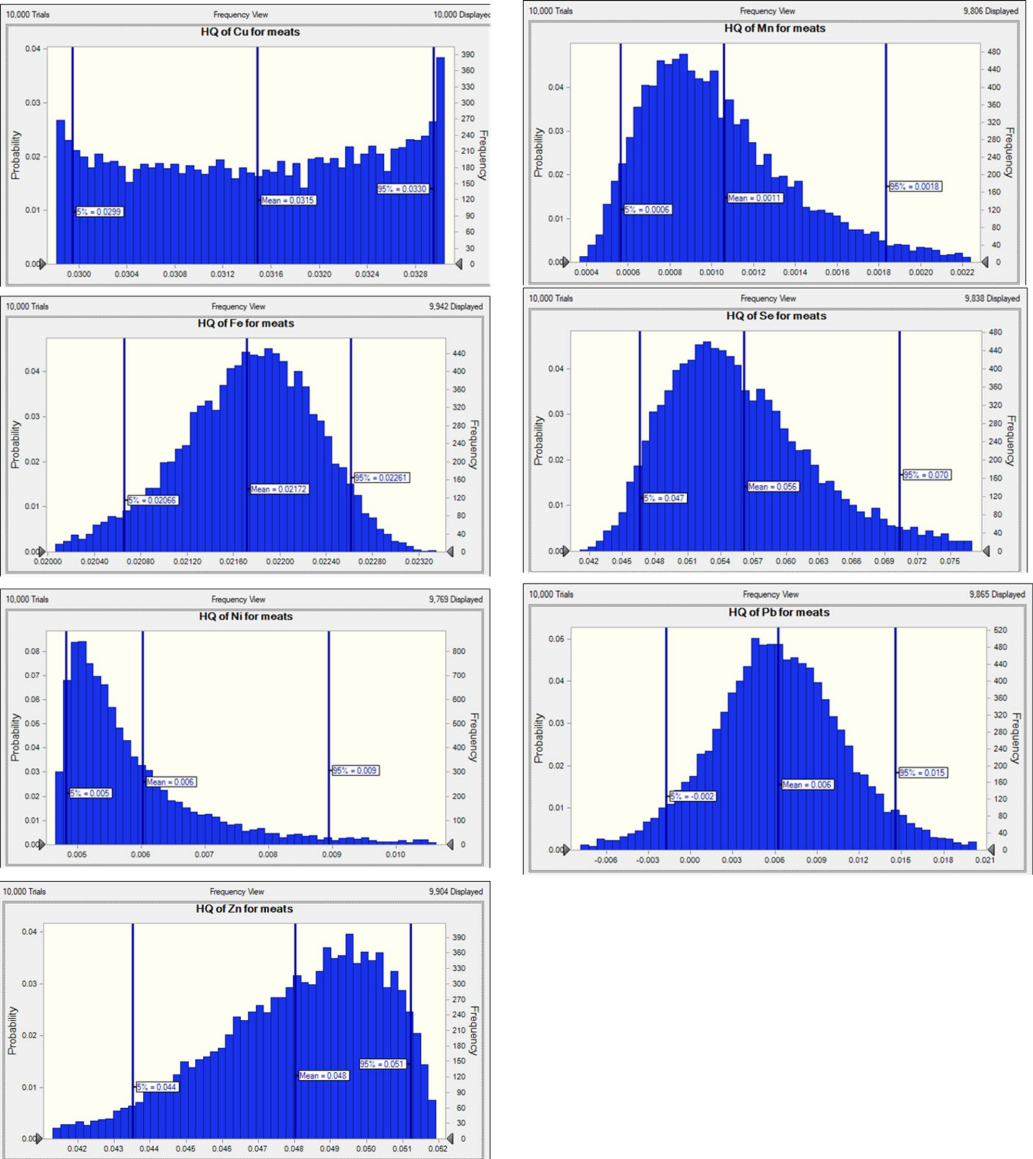
Target hazard quotient (THQ) distribution for Cu, Mn, Fe, Se, Ni, Pb, and Zn by meat consumption by Monte Carlo simulation.

**Fig. 3:**
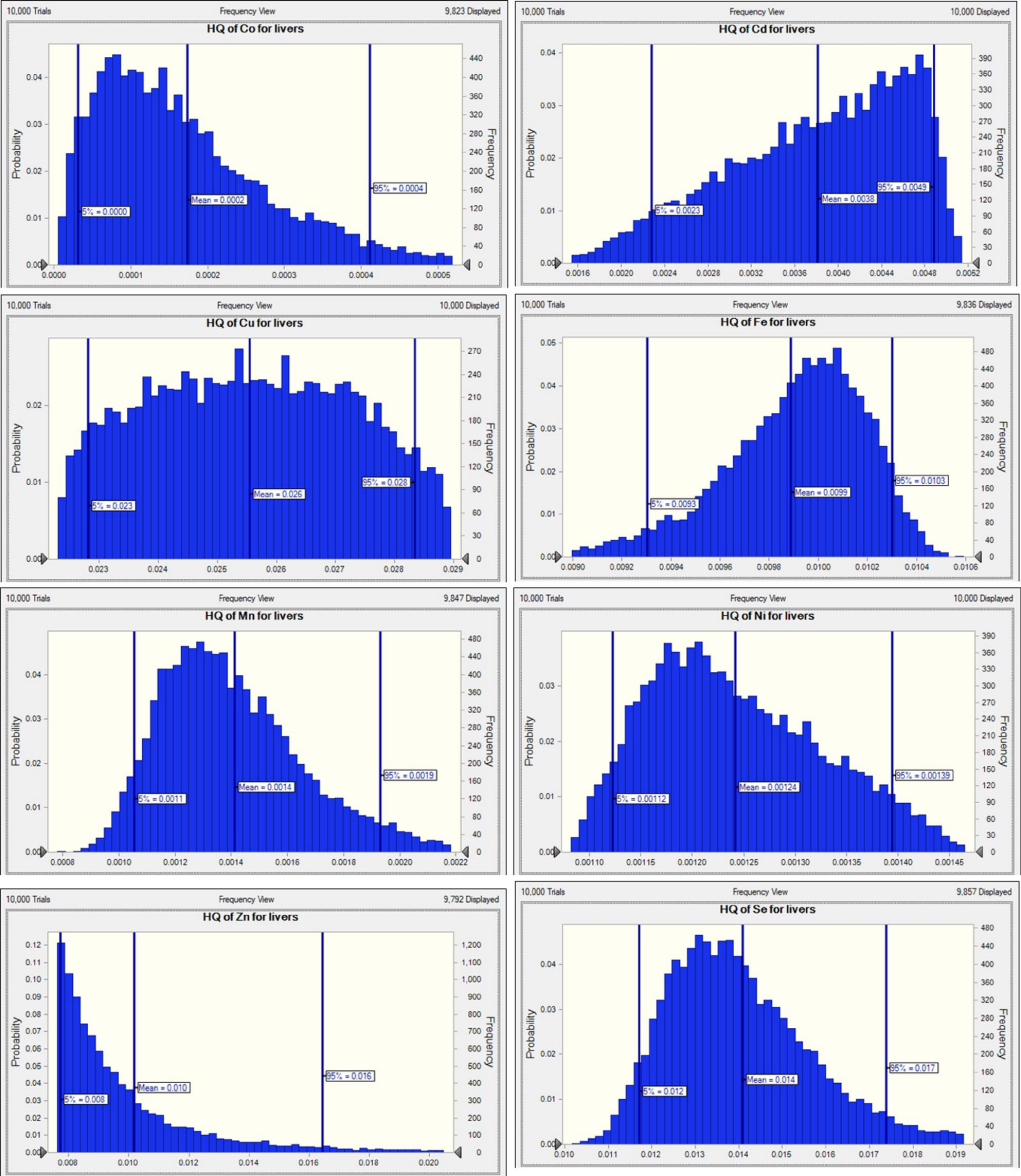
Target hazard quotient (THQ) distribution for Co, Cd, Cu, Fe, Mn, Ni, Zn, and Se through liver consumption by Monte Carlo simulation.

The risk of non-carcinogenic effects from the toxic and essential trace element in consumers was assessed by calculating the HQ value. The findings from the analysis were used to compute the overall and maximum HQ levels for toxic and essential trace elements in the adult population, as illustrated in Figs. 2 and 3. This value is considered a suitable parameter for assessing the risks associated with consuming toxic and essential trace elements from contaminated food. The THQ is defined as the ratio of the observed amount of a contaminant to the reference RfD for the substance^80^. The presence of a high level of HQ > 1 in toxic and essential trace elements signifies a potential health hazard if food is contaminated with these elements. Conversely, HQ < 1 suggests that the associated health risk is negligible^81^. According to Table 4, The THQ values revealed that, in liver samples, the elements followed the order: Cu > Se > Zn > Fe > Pb > Cd > Mn > Ni > Co, indicating that Cu posed the highest potential health risk. In contrast, the order in meat samples was Se > Zn > Cu > Fe > Pb > Ni > Mn, suggesting Se contributed most to the total hazard in meat.

**Table 4:** International lifetime cancer risk (ILCR), hazard quotient (HQ), and hazard index (HI) specific to consumers of small ruminant carcasses slaughtered in Khorramabad, Iran.

	ILCR		HQ	
	Liver	Muscle	Liver	Muscle
Cd	7.330667E-5	ND	0.0049	ND
Ni	2.537057E-5	1.596439E-4	0.00139	0.009
Pb	1.932756E-7	4.947834E-7	0.006	0.015
Co	-	-	0.0004	ND
Se	-	-	0.017	0.070
Fe	-	-	0.0103	0.02261
Mn	-	-	0.0019	0.0018
Cu	-	-	0.028	0.0330
Zn	-	-	0.016	0.051
HI			0.09	0.20

CR > E-4 is unacceptable; E-6 < CR < E-4 is acceptable; CR < E-6 is neglectable.

**Fig. 4:**
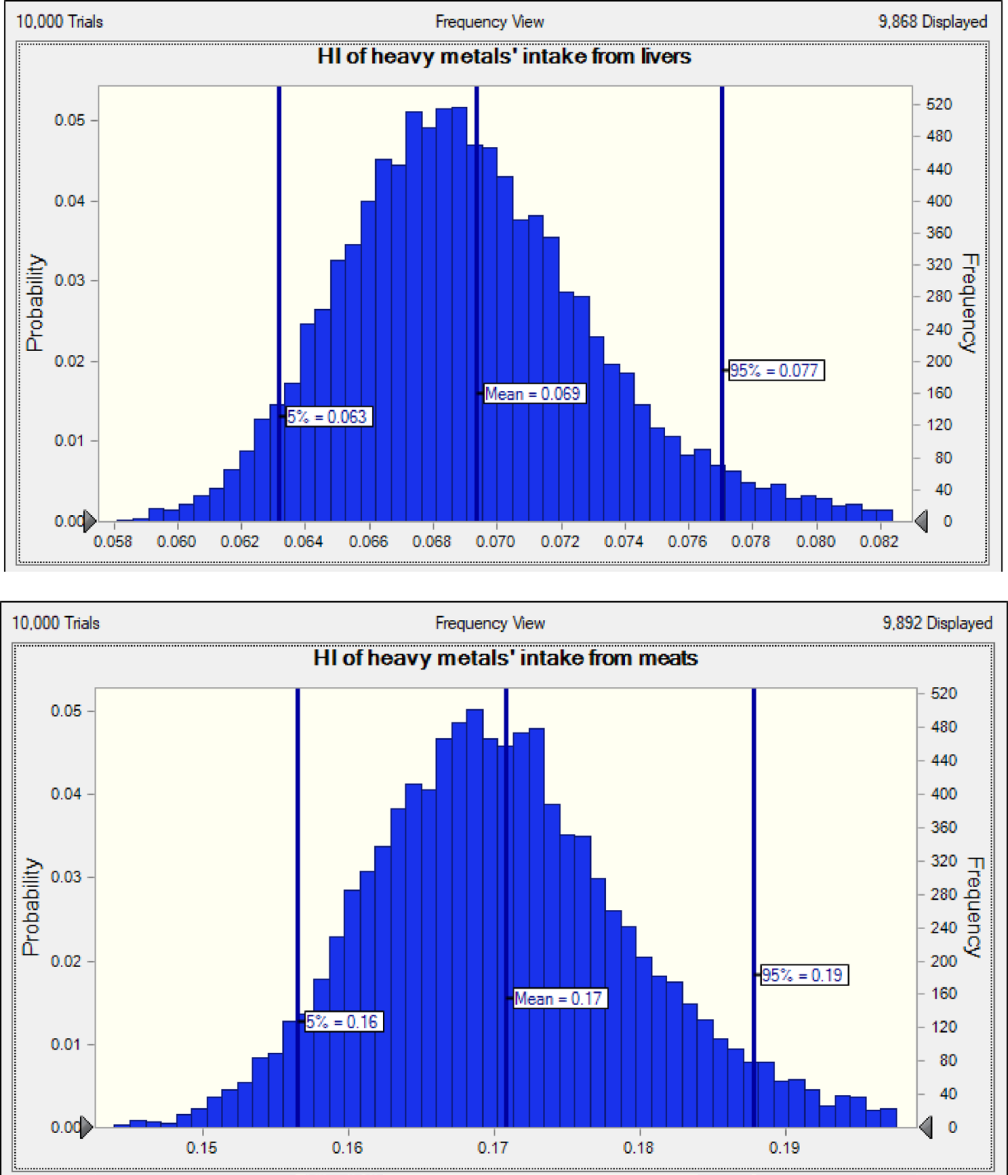
The Hazard Index (HI) distribution for Cu, Fe, Mn, Ni, Pb, Se, and Zn through meat and liver consumption by Monte Carlo simulation.

The Monte Carlo simulation results (Figs. 2 and 3) indicate that the mean HQ values for heavy metals in meat, including Cu (0.0315), Mn (0.0018), Fe (0.0202), Se (0.0560), Ni (0.0064), Pb (0.0056), and Zn (0.0488), are all significantly below the safety threshold of 1, suggesting negligible non-carcinogenic health risks from these elements even under high-exposure scenarios. Additionally, the 95th percentile HQ values for these metals further confirm their low risk profiles, with Se having the highest value at 0.0636 and Mn the lowest at 0.0024. In liver samples, the HQ distributions for Co and Cd are centered around 0.0002 and 0.0004, respectively, while Cu, Mn, Zn, Ni, Fe, and Se show similarly low values, indicating minimal exposure risks. HI was calculated for each tissue in small ruminants in adults (Fig. 4; Table 4). HI was numerically less than one for meat and liver of goat and sheep, and liver was calculated to be less than meat (Fig. 4). There was no apparent risk to consumers from the current study of the meat and liver of small ruminants, which is consistent with previous studies by Wang, et al^82^..

Long-term consumption of harmful metals found in edible offal and meat can cause toxicity due to their accumulation in the body. Therefore, the research recommended regular monitoring of these toxic metals in the meat and liver of animals intended for human consumption in that area. This study suggested that it is important to monitor chemical contaminants in meat and edible offal to maintain good health. However, in recent years, there has been a notable decline in the consumption of meat and meat products in Iran. This reduction poses challenges in accurately estimating the actual exposure to metal contaminants through meat.

### Carcinogenic risk

**Fig. 5:**
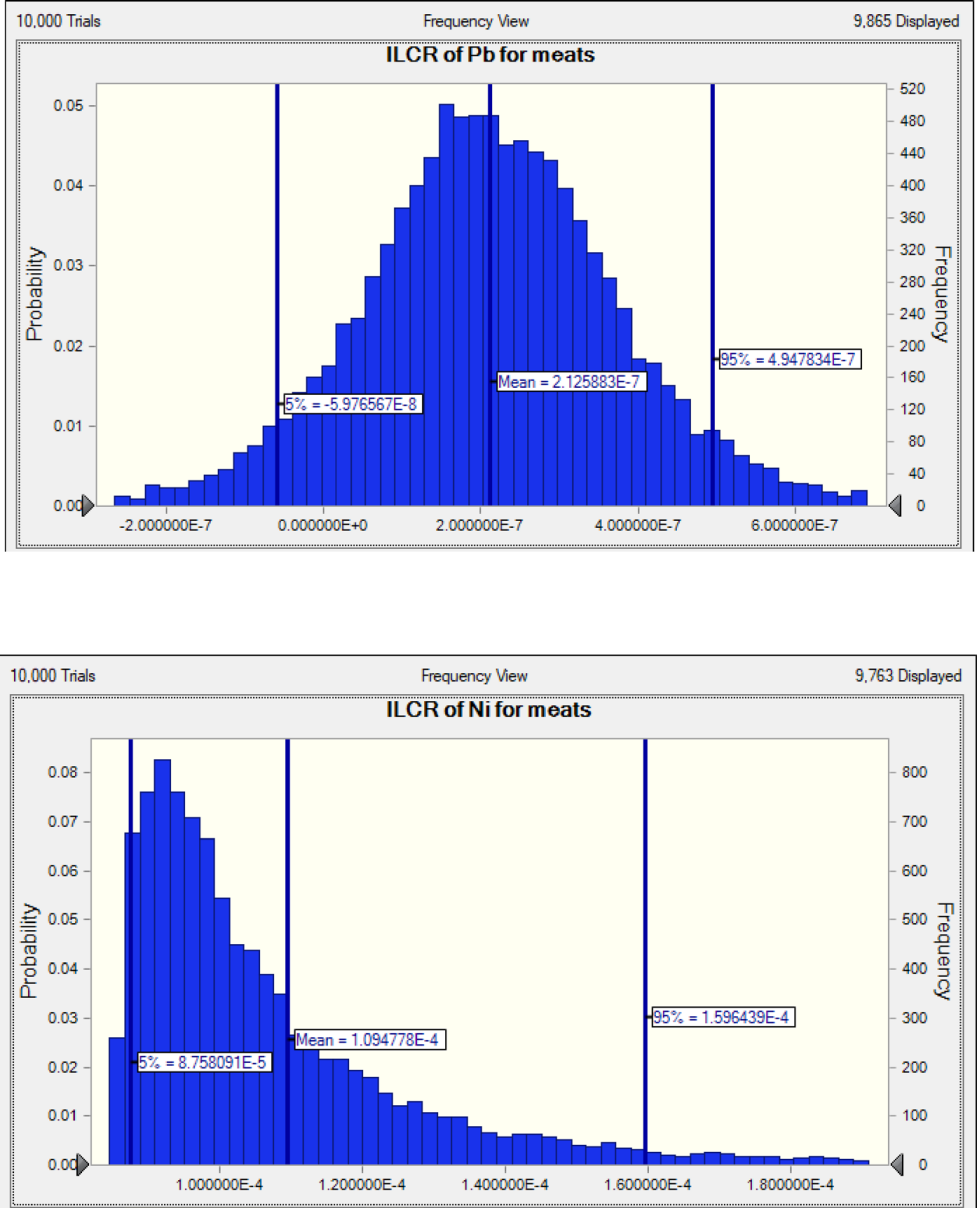
Incremental lifetime cancer risk (ILCR) of Ni, and Pb through meat consumption.

**Fig. 6:**
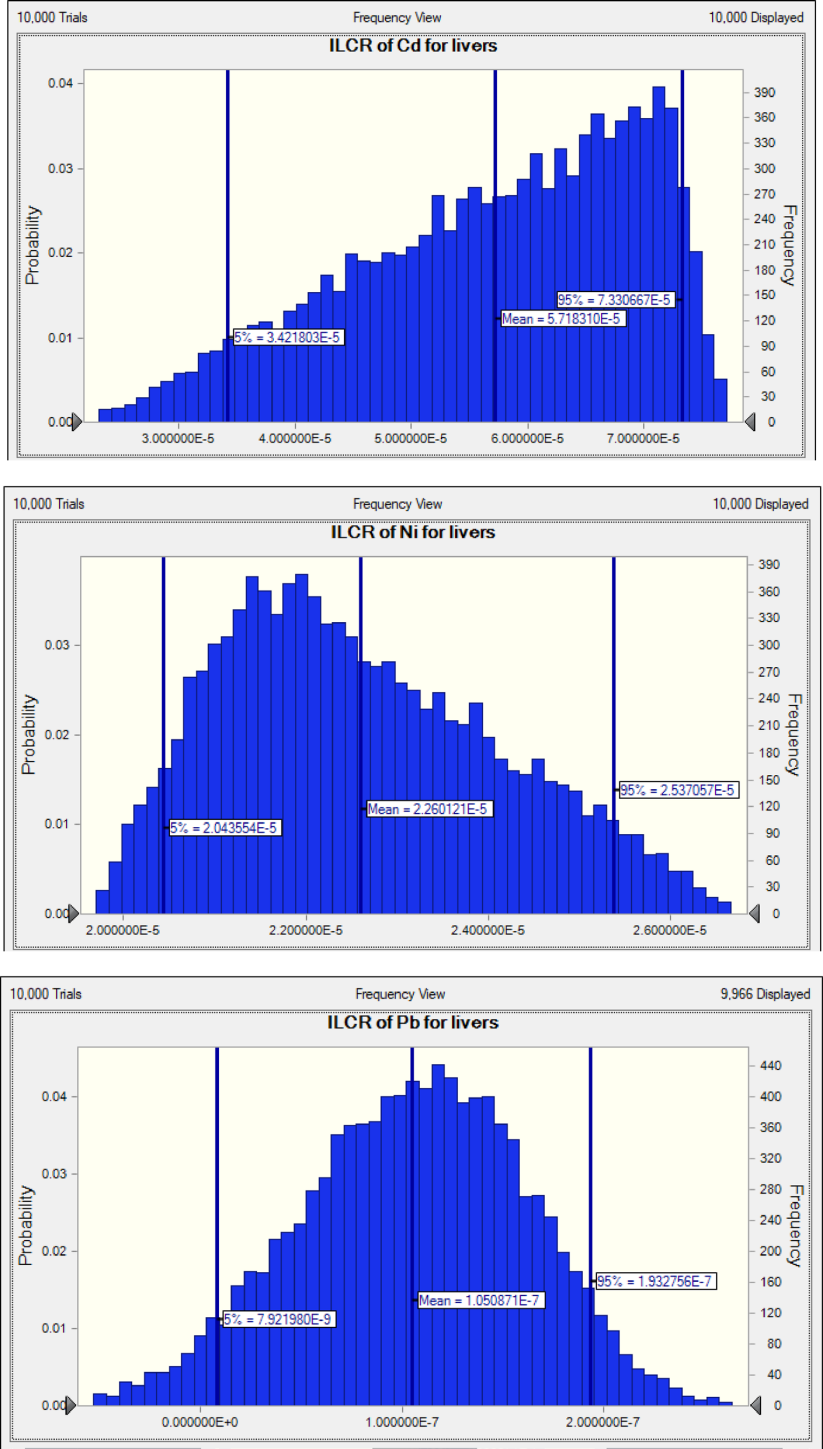
Incremental lifetime cancer risk (ILCR) of Cd, Ni, and Pb through liver consumption.

The US EPA specifies a safe cancer risk threshold of less than a lifetime exposure of 10^− 6^ (IR < 10^− 6^). In contrast, the established threshold for concern is an IR greater than 10⁻⁴. The cancer risk index calculations were primarily based on exposure to Pb, Cd, and Ni, derived from maximum consumption levels. This approach was necessitated by the lack of oral cancer slope factors for other metals classified as carcinogenic by the IARC. The findings from the examination of toxic elements in both the liver and meat (Table 4; Figs. 5 and 6) show LCD values ranging from 1.596 × 10^− 4^ to 1.93 × 10^− 7^. The ILCR values for liver and meat exceeded 1.0 × 10^− 4^, suggesting that dietary consumption presents a notable carcinogenic risk to the population. For Ni, the ILCR values in meat and liver were between 2.537 × 10^− 5^ and 1.596 × 10^− 4^, indicating some level of carcinogenic risk. The two primary routes of exposure to heavy metals involve growing plants in metal-contaminated soils or irrigating with contaminated water, which can result in animals grazing on pastures with high levels of heavy metals, leading to bioaccumulation. However, in a report published in late 2020, the European Food Safety Authority (EFSA) stated that Ni is no longer classified as a carcinogenic hazard when present in food^22,83^. Consequently, it can be inferred that Ni does not pose a significant health risk through dietary exposure.

The ILCR value for Cd in meat was zero, as it was not detected; however, the cumulative carcinogenic risk from Cd in the liver within the residents’ daily diet was under 1.0 × 10^− 4^. Furthermore, the 95th percentile ILCR values for Pb in meat and liver were 1.93 × 10^− 7^ and 4.95 × 10^− 7^, respectively, none of which surpassed the maximum acceptable threshold of 1.0 × 10^− 4^.

However, the findings of this study indicate a moderate carcinogenic risk associated with Ni and elevated levels of Zn and Pb in the tissues of small ruminants, which could induce oxidative stress and compromise immune function in consumers over time. To mitigate such negative health impacts in livestock and potentially reduce the bioavailability of these metals, future research could explore the use of natural dietary supplements with antioxidant and immunomodulatory properties. In this context, the application of dietary hemp (*Cannabis sativa*L.) and its cannabinoids present a promising avenue. A recent review by Hassan, et al^84^. highlighted the significant potential of cannabinoids, particularly cannabidiol (CBD), to act as powerful antioxidants and modulate the immune response in animals. Investigating the inclusion of hemp-based feed additives could be a strategic approach to enhance the resilience of animals to environmental pollutants and improve the overall safety profile of meat products.

### Source identification

**Fig. 7:**
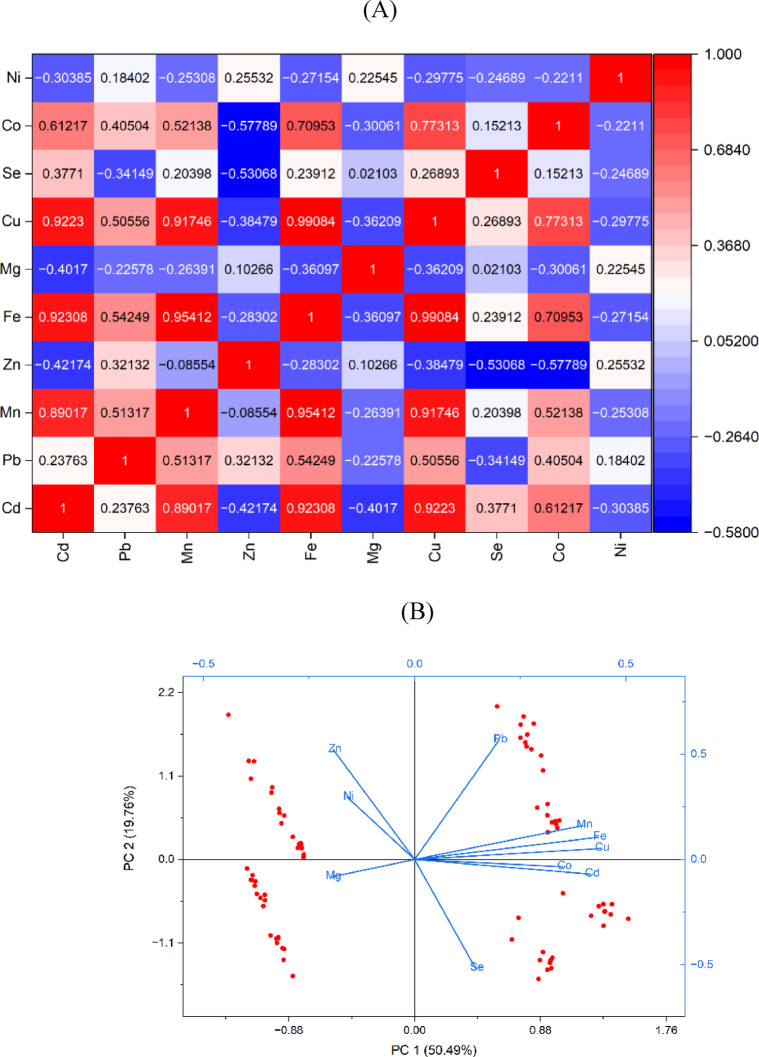

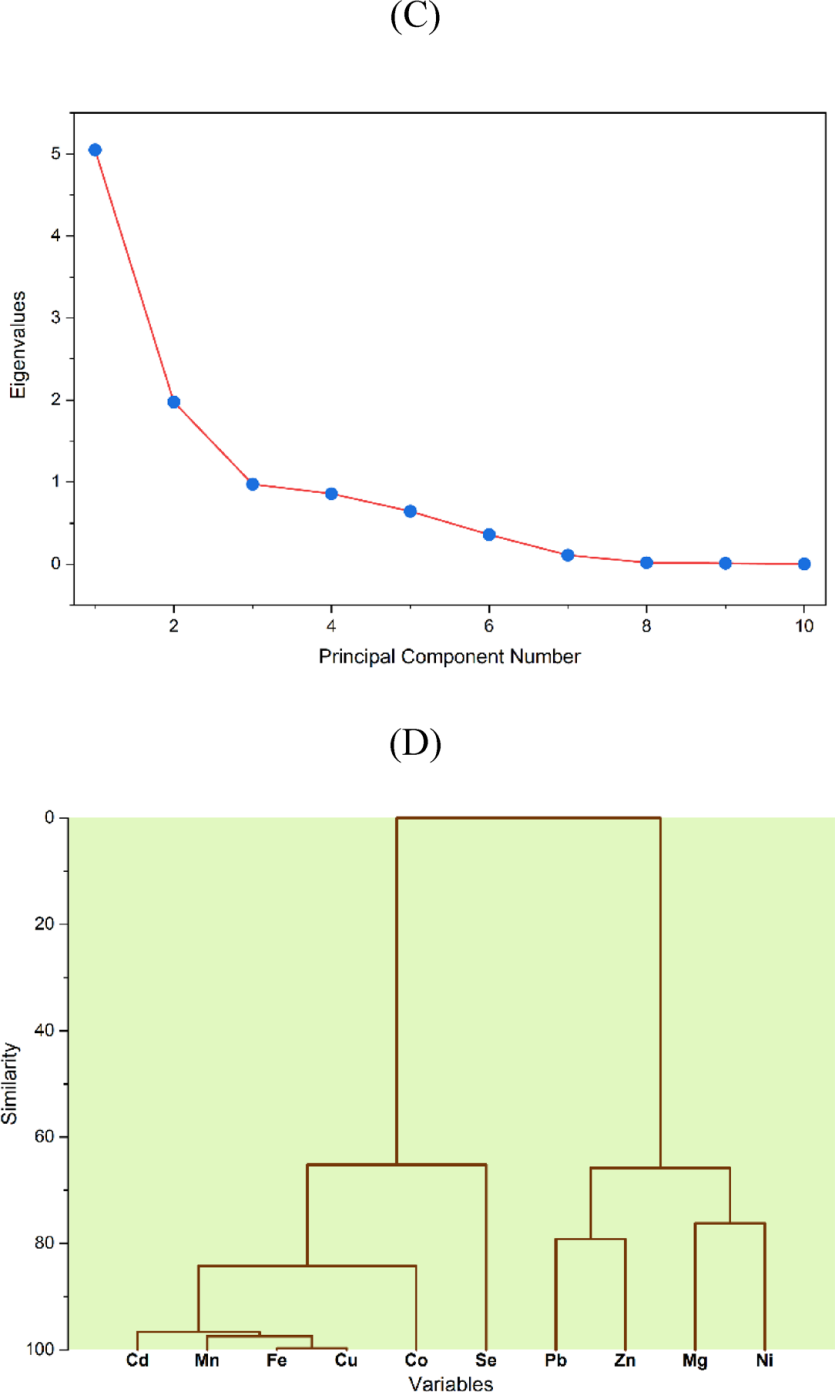
Overview of PCC correlation (**A**), PCA parameters (**B**), scree plot (**C**), Dendrogram demonstration of HCA clusters (**D**).

To investigate the distribution patterns and relationships between heavy metals and essential elements in the samples, PCC was performed. PCC analysis (Fig. 7A) assesses linear relationships between element concentrations, where strong positive correlations indicate interdependence and potential common sources. PCC is a widely used tool for assessing variable relationships, with an r value > 0.7 indicating strong correlation^85^. High correlations were observed in pairs such Cd-Mn, Cd-Fe, Mn-Fe, Cu-Co, and Co-Fe, suggesting that these elements likely have a common origin or accumulate through related pathways. Moderate correlations, typically defined as values between 0.5 and 0.7, suggest minor but meaningful relationships that may reflect overlapping or shared sources^86^. In this study, such correlations were observed between Co-Cd (*r* = 0.61), Mn-Co (*r* = 0.52), and Fe-Pb (*r* = 0.54). Thus, PCC provides an initial understanding of potential common sources for elements, which are further confirmed through PCA and HCA.

The biplot of the first two principal components is shown in Fig. 7B. The first component (PC1) and the second component (PC2) explained 50.49% and 19.76% of the total variance, respectively, together accounting for approximately 70.25% of the overall variability. PC1 demonstrated significant positive loadings for Cu, Mn, Fe, and Cd, suggesting a common source or geochemical association. PC2 exhibited strong positive loadings for Pb, Zn, and Ni, indicating a likely shared origin among these elements. Interestingly, Zn presented a negative loading on PC1 and showed strong inverse correlations, particularly with Se, implying a distinct source or pathway. Furthermore, a moderate negative correlation was observed between Se and Co.

Finally, HCA complemented PCC and PCA by grouping elements into clusters based on similarity, which are visually represented in a dendrogram. In Fig. 7D, elements such as Cu, Cd, Fe, Co, and Mn are grouped together, indicating a common source. Overall, the primary source of elements in the analyzed samples appears to be a ‘terrestrial factor,’ as reflected by PC1 and previously noted by Moghtaderi, et al^87^. in studies of industrial regions. These metals readily bioaccumulate in plants^88^ and can subsequently transfer to ruminants grazing in contaminated areas, often serving as indicators of local environmental pollution.

Another cluster of elements, including Pb, Ni, and Zn, can be classified as anthropogenic because they show a strong association with human activities such as industrial emissions, transportation, and agricultural practices that involve machinery, pesticides, and fertilizers^6^. These cross-validated results highlight the need for focused monitoring and control measures to improve pollution management practices and to help protect public health and food safety in the region.

### Limitations

This study has several limitations. First, there is no set maximum limit for heavy metals in goat meat and liver, which makes direct comparisons difficult. Because different species accumulate metals in different ways, future regulations should consider separate threshold values for various livestock species. This study also did not take into account possible confounding factors such as age, environmental exposure, and seasonal changes, which could affect metal levels. Only fresh meat and liver samples were analyzed, so the results may not represent heavy metal levels in other edible products or processed forms. Future studies should address these limitations to improve representativeness and reduce uncertainty.

## Conclusion

This study demonstrated significant differences in the mean concentrations of toxic and essential trace elements between males and females and between sheep and goats in both meat and liver tissues. Cd, Pb, Co, Cu, and Fe were consistently higher in liver compared to meat. Although the average concentrations of Cd and Pb in most samples were below the maximum residue limits (except Mn, Ni, Pb, and Zn), the long-term accumulation of these elements may pose health risks to consumers. Notably, the concentration of Ni exceeded the permissible limit. Health risk assessment through THQ, HI, and ILCR indicated that metal residues in small ruminant tissues may pose moderate human health risks. Multivariate statistical analyses (PCC, PCA, HCA) showed distinct geogenic (terrestrial) sources for Cd, Mn, Fe, Cu, and Co, while Pb, Ni, and Zn were linked primarily to anthropogenic activities.

The findings reveal a concerning trend of heavy metal bioaccumulation in organisms, raising issues regarding potential toxicity and human exposure through the food chain. This underscores the urgent need for coordinated efforts to assess and address the broader impacts of heavy metal contamination. Future research should focus on understanding the mechanisms of bioaccumulation to develop specific mitigation strategies. Additionally, exploring natural dietary supplements with antioxidant and immune-modulating properties could be a promising method for reducing metal bioavailability in livestock and enhancing meat safety. Strengthening the monitoring of metal contamination in soils and agricultural products, along with implementing sustainable farming practices, is essential for protecting ecosystems and safeguarding public health.

## Supplementary Information

Below is the link to the electronic supplementary material.

Supplementary Material 1

## Data Availability

The data that support the findings of this study are available from the corresponding author upon reasonable request.
