# Supplementary material for: Potentially toxic metals in small ruminant tissues: multivariate analysis and health risk assessment via Monte Carlo simulation
Source: Sci Rep. 2025 Dec 27;16:3704. doi: 10.1038/s41598-025-33838-2 (PMC12852769; doi:10.1038/s41598-025-33838-2)

**Supplementary Material:**

**Health Risk Assessment of Heavy Metals Exposure in Small Ruminant Tissue through Monte Carlo Simulation: A Cross-sectional Study from Iran**

Alireza Fathi-Beyranvand^a^, Azadeh Rashidimehr^a*^, Elaheh Askari^b*^, Fatemeh Esfarjani^c^, Fatemeh Mohammadi-Nasrabadi^c^

*^a^* *Microbiology and Food Hygiene Department, Faculty of Veterinary Medicine, Lorestan University, Khorramabad, Iran*

*^b^ Nutritional Health Research Center, School of Health and Nutrition, Lorestan University of Medical Sciences, Khorramabad, Iran*

*^c^ Food and Nutrition Policy and Planning Research Department, Faculty of Nutrition Sciences and Food Technology, National Nutrition and Food Technology Research Institute, Shahid Beheshti University of Medical Sciences, Tehran, Iran*

**List of Tables: Table S1:** LOD, LOQ, Precision, Matrix Effect, Recovery, and Specificity of the for ICP-OES Determination of Essential and Toxic Metals

**Table S1:** Recovery Results Obtained from Tissue Samples

List of Figures:

**Figure S1** Calibration Curve for Cadmium (Cd)

**Figure S2** Calibration Curve for Cobalt (Co)

**Figure S3** Calibration Curve for Copper (Cu)

**Figure S4** Calibration Curve for Iron (Fe)

**Figure S5** Calibration Curve for Manganese (Mn)

**Figure S6** Calibration Curve for Nickel (Ni)

**Figure S7** Calibration Curve for lead (Pb)

**Figure S8** Calibration Curve for Selenium (Se)

**Figure S9** Calibration Curve for Zinc (Zn)

**Figure S10** Calibration Curve for Magnesium (Mg)

**Table S1:**

| **Metal** | **LOD (ppb)** ^*^ | **LOQ (ppb)**^*^ | **Intraday Precision** | **Interday Precision (%RSD)** | **Matrix Effect (%)** | **Specificity^**^** |
| --- | --- | --- | --- | --- | --- | --- |
| Pb | 0.942 | 2.854 | 0.661 | 0.87 | 1.15 | Verified |
| Co | 0.160 | 0.485 | 1.971 | 2.778 | 0.64 | Verified |
| Cu | 0.904 | 2.739 | 0.453 | 0.239 | 0.13 | Verified |
| Mn | 0.036 | 0.109 | 0.265 | 1.202 | 1.4 | Verified |
| Se | 6.638 | 20.12 | 1.21 | 0.565 | 0.39 | Verified |
| Ni | 0.749 | 2.270 | 2.207 | 0.160 | 0.37 | Verified |
| Cd | 0.127 | 0.385 | 1.552 | 1.12 | 0.28 | Verified |
| Fe | 0.225 | 0.682 | 0.437 | 0.329 | 1.82 | Verified |
| Mg | 1.0007 | 3.032 | 0.338 | 2.133 | 2.36 | Verified |
| Zn | 0.173 | 0.524 | 0.567 | 0.256 | 0.54 | Verified |

^*^ LOD = 3.3 × σ / S; LOQ = 10 × σ / S, where σ is the standard deviation of the response and S is the slope of the calibration curve.

^**^ Specificity was verified for all analytes by the absence of significant matrix interferences, consistent precision, and the use of interference-free analytical wavelengths, according to method validation and literature standards.

**Table S2:**

| **Metal** | **Spike amount (ppb)** | **Measured amount (ppb)±SD^*^** | **Recovery (%)** |
| --- | --- | --- | --- |
| Pb | 500 | 493.3±0.07 | 98.6698.66 |
| Co | 500 | 511.85±0.03 | 102.37 |
| Cu | 500 | 504.45±0.02 | 100.89 |
| Mn | 500 | 516.8±0.02 | 103.36 |
| Se | 500 | 497.7±0.05 | 99.54 |
| Ni | 500 | 513.7±0.02 | 102.74102.74 |
| Cd | 500 | 517.15±0.01 | 103.43 |
| Fe | 500 | 479.45±0.04 | 95.89 |
| Mg | 500 | 499.55±0.04 | 99.91 |
| Zn | 500 | 508.6±0.03 | 101.72 |

*SD=Standard deviation

**Figure S1**


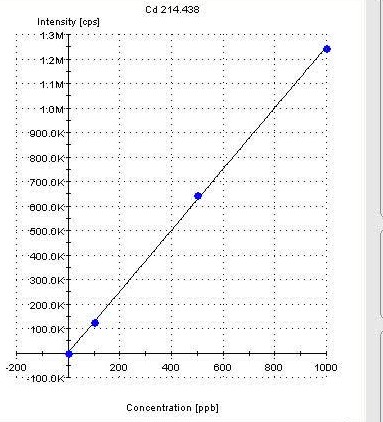


**Figure S2**


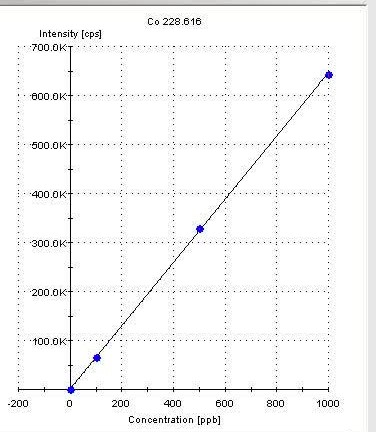


**Figure S3**


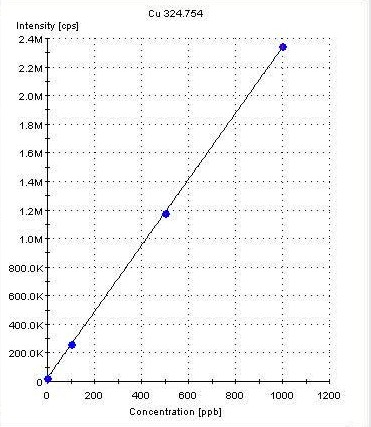


**Figure S4**


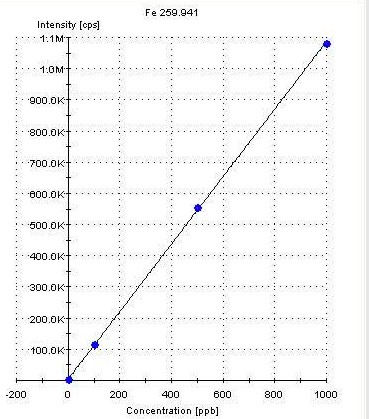


**Figure S5**


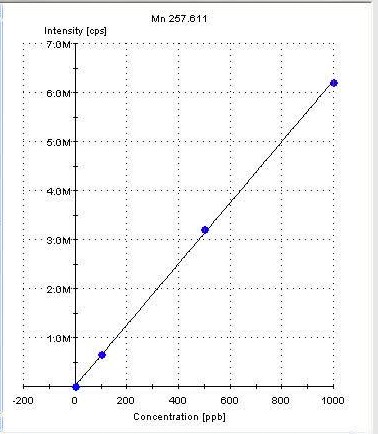


**Figure S6**


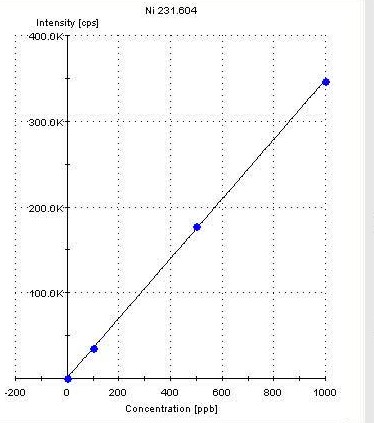


**Figure S7**


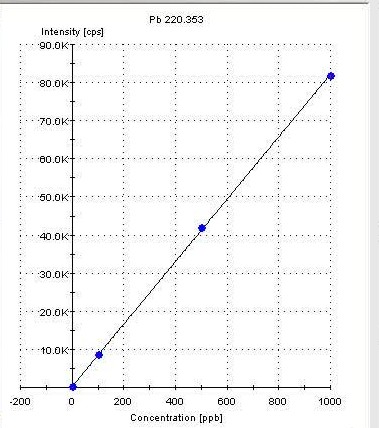


**Figure S8**


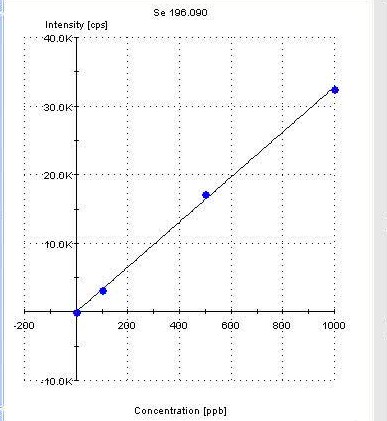


**Figure S9**


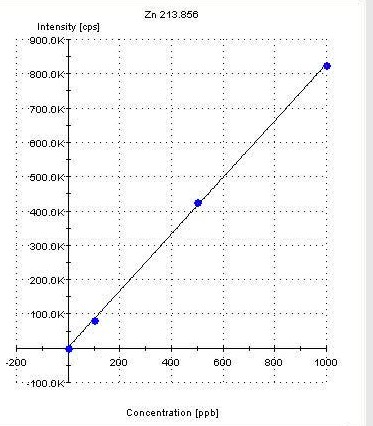


**Figure S10**


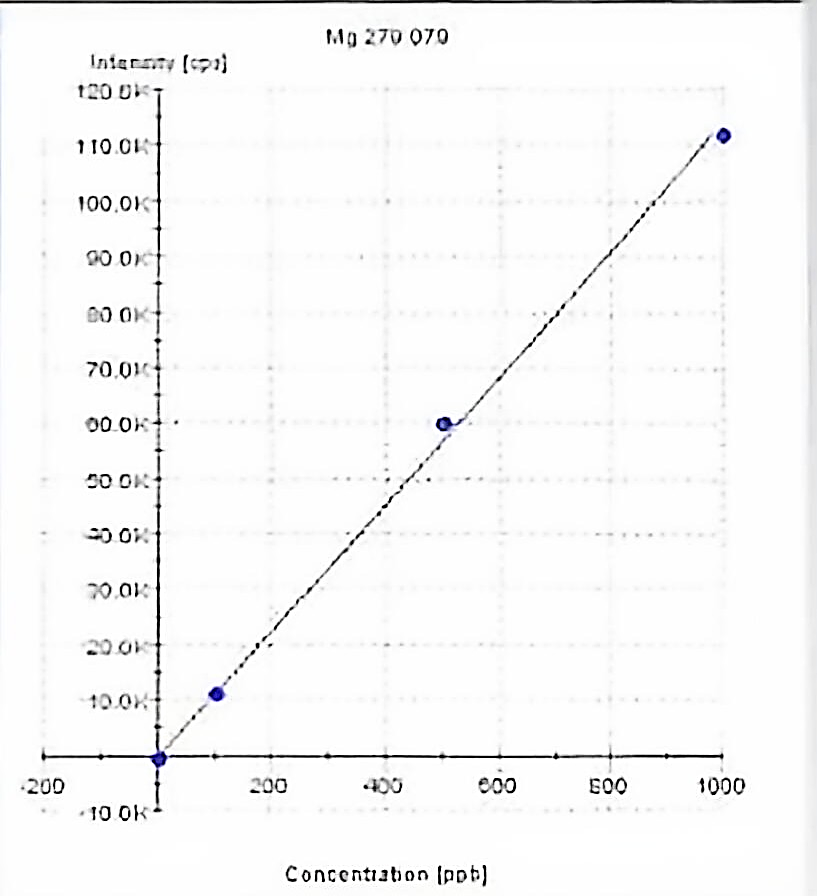

Supplement: Supplementary file 1 — Supplementary Material 1 [file 41598_2025_33838_MOESM1_ESM.docx]
